# Estimating Uncertainty of Geographic Atrophy Segmentations with Bayesian Deep Learning

**DOI:** 10.1016/j.xops.2024.100587

**Published:** 2024-07-24

**Authors:** Theodore Spaide, Anand E. Rajesh, Nayoon Gim, Marian Blazes, Cecilia S. Lee, Niranchana Macivannan, Gary Lee, Warren Lewis, Ali Salehi, Luis de Sisternes, Gissel Herrera, Mengxi Shen, Giovanni Gregori, Philip J. Rosenfeld, Varsha Pramil, Nadia Waheed, Yue Wu, Qinqin Zhang, Aaron Y. Lee

**Affiliations:** 1 Department of Ophthalmology, University of Washington, Seattle, Washington; 2 The Roger and Angie Karalis Retina Center, Seattle, Washington; 3 Topcon Healthcare, Oakland, New Jersey; 4 Department of Bioengineering, University of Washington, Seattle, Washington; 5 Carl Zeiss Meditec, Inc., Dublin, California; 6 Twenty Twenty Therapeutics, LLC, San Francisco, California; 7 Department of Ophthalmology, Bascom Palmer Eye Institute (BPEI), University of Miami Miller School of Medicine, Miami, Florida; 8 Tufts University School of Medicine, Boston, Massachusetts; 9 New England Eye Center, Tufts New England Medical Center, Boston, Massachusetts

**Keywords:** Age-Related macular degeneration (AMD), Bayesian deep learning, Geographic atrophy (GA), Model uncertainty, OCT

## Abstract

**Purpose:**

To apply methods for quantifying uncertainty of deep learning segmentation of geographic atrophy (GA).

**Design:**

Retrospective analysis of OCT images and model comparison.

**Participants:**

One hundred twenty-six eyes from 87 participants with GA in the SWAGGER cohort of the Nonexudative Age-Related Macular Degeneration Imaged with Swept-Source OCT (SS-OCT) study.

**Methods:**

The manual segmentations of GA lesions were conducted on structural subretinal pigment epithelium en face images from the SS-OCT images. Models were developed for 2 approximate Bayesian deep learning techniques, Monte Carlo dropout and ensemble, to assess the uncertainty of GA semantic segmentation and compared to a traditional deep learning model.

**Main Outcome Measures:**

Model performance (Dice score) was compared. Uncertainty was calculated using the formula for Shannon Entropy.

**Results:**

The output of both Bayesian technique models showed a greater number of pixels with high entropy than the standard model. Dice scores for the Monte Carlo dropout method (0.90, 95% confidence interval 0.87–0.93) and the ensemble method (0.88, 95% confidence interval 0.85–0.91) were significantly higher (*P* < 0.001) than for the traditional model (0.82, 95% confidence interval 0.78–0.86).

**Conclusions:**

Quantifying the uncertainty in a prediction of GA may improve trustworthiness of the models and aid clinicians in decision-making. The Bayesian deep learning techniques generated pixel-wise estimates of model uncertainty for segmentation, while also improving model performance compared with traditionally trained deep learning models.

**Financial Disclosures:**

Proprietary or commercial disclosure may be found in the Footnotes and Disclosures at the end of this article.

Clinical diagnoses in medicine are mostly probabilistic. Clinicians construct a differential diagnosis through the evaluation of potential medical conditions and their probabilities. As more information is collected, the final diagnosis is made with varying degrees of certainty. In contrast, deep learning models, which are increasingly incorporated into medical practice, yield categorical diagnostic outputs without corresponding confidence measures. This shortcoming is a result of a typical supervised deep learning setup in which a deep neural network is trained to map inputs to labels by minimizing a loss function. After training, the model's weights are fixed. When the trained model makes predictions on new data, it will predict a categorical outcome. This categorical outcome prevents any nuanced insight into the model's decision-making and deprives users of understanding the degree of confidence behind each prediction. Traditional deep learning's deterministic nature poses a significant challenge for understanding confidence as it bypasses the essential aspect of expressing uncertainty when a clinical diagnosis is made. This is relevant for fields such as clinical medicine where it is paramount to understand the relative certainty in a particular diagnosis or describing the borders of a lesion.

Numerous efforts have been undertaken to shed light on the inner workings of artificial intelligence (AI) systems, seeking to bridge the gap between their decision-making processes and human understanding.^1^ These approaches fall into 2 main camps. First, there are explainability methods, such as saliency maps and gradient-weighted class activation mapping,^2^^,^^3^ which highlight the pertinent areas of the input images for model prediction, thereby providing visual and intuitive insights for humans. While these visualization methods shed light on how the AI made its decision, they do not explicitly address the confidence of these predictions.^4^

The other camp focuses on estimating the probability of the predicted outcome and the degree of uncertainty in the prediction. There are 2 types of uncertainty models: aleatoric and epistemic uncertainty.^5^ Aleatoric uncertainty is uncertainty inherent in the data, such as label noise and image distortions. In contrast, epistemic uncertainty is the uncertainty intrinsic to the model, such as model initializations and the balance or imbalance between model complexity and the amount of training data. While aleatoric uncertainty can be modeled with techniques such as using maximum a posteriori inference with loss functions, epistemic uncertainty can be modeled by perturbing the characteristics of the model. These types of uncertainty are particularly relevant in clinical medicine where both the data and the models affect the performance. When utilizing machine learning models in clinical medicine, where data or aleatoric uncertainty cannot always be controlled, it is imperative to measure and minimize epistemic uncertainty. Once measured, uncertainty can be integrated into AI model predictions to offer a more comprehensive understanding of the AI system behavior.

Uncertainty is primarily modeled through a field of deep learning called Bayesian deep learning. Bayesian deep learning uses probability to model the posterior distribution of the model weights but is computationally expensive. Due to these difficulties, numerous methods have been used to approximate Bayesian deep learning by using modified deterministic deep learning models. Two representative Bayesian methods are (1) training a model with Monte Carlo dropout and (2) training an ensemble of models. Training a model with dropout allows sampling of multiple outputs from the same input at inference time to estimate uncertainty.^6^ Training an ensemble of models with different initial weights allows comparison of the output from each model at inference time to estimate uncertainty.^7^ In either case, the same input is run through a model multiple times with perturbations of its weights to produce a Monte Carlo approximation of the posterior distribution over the model weights.

We chose to use geographic atrophy (GA) as a practical example to demonstrate how uncertainty can be modeled, displayed and used. Geographic atrophy involves atrophy of the outer retinal layers, retinal pigment epithelium, and choriocapillaris that occurs in the later stages of dry age-related macular degeneration. Geographic atrophy is progressive and can impact vision, causing irreversible vision loss and legal blindness as the lesions extend into the fovea.^8^ Heterogeneity in the appearance of GA lesions adds challenge to their identification.^9^ In the context of GA segmentation, visualizing uncertainty in conjunction with predictions may be preferable over categorical pixel classification, as it provides clinicians with a nuanced understanding of a model's confidence level in the presence of GA.

In this study, we investigated 2 approximate Bayesian deep learning techniques, Monte Carlo dropout and ensemble, to assess the uncertainty of GA semantic segmentation. Monte Carlo dropout has been demonstrated to be an approximation of minimizing the Kullback-Leibler divergence.^6^ We compared Monte Carlo dropout against ensembles, as prior research^10^ found that ensembles had superior performance over Kullback-Leibler-minimizing.^6^ We visualized the pixel-wise uncertainty, compared relative model performance, and investigated the relationship between uncertainty and model performance. Our analysis showed the degree of uncertainty associated with each pixel, which can help clinicians interpret deep learning segmentation outputs with caution and facilitate decision-making.

## Methods

### Data Acquisition and Ethics

This was a retrospective study of prospectively collected data from the SWAGGER cohort of the Nonexudative Age-Related Macular Degeneration Imaged with Swept-Source OCT (SS-OCT) extension study (ClinicalTrials.gov id NCT04469140) and from the New England Eye Center at Tufts Medical Center. This study was approved by the Institutional Review Board of Tufts Medical Center and was conducted in accordance with the Declaration of Helsinki. Informed consent was obtained from all participants in the SWAGGER study. Informed consent was exempted by the Institutional Review Board for the New England Eye Center data because of the study’s retrospective design.

### Data Preparation

A total of 126 SS-OCT scans were taken from 126 eyes (87 patients) from 3 clinical study sites (sites 1, 2, and 3), and pseudo-color en face images were generated from these using a previously described algorithm that included the main SS-OCT features of GA as training inputs: hypertransmission defects detected on the subretinal pigment epithelium en face slab (64 μm–400 μm beneath Bruch’s membrane), regions of retinal pigment epithelium loss, and loss of retinal thickness.^11^ Next, a grader at each of the study sites manually segmented the GA lesions in the structural subretinal pigment epithelium en face images. Graders (M.S., G.H.) were trained before performing annotation and followed the consensus grading protocol previously published by Liu et al.^12^ The resulting manually segmented lesions and accompanying images were split into training and validation sets. An independent test set containing 225 unique scans from 72 eyes (51 patients) was also collected from site 1, and each of these images was segmented by 2 masked graders (M.S., G.H.).

### UNet Model Architecture

All the models described in this paper were built upon the same UNet architecture, which was similar to the original U-Net architecture.^13^ It consisted of a contractive path, in which the feature maps were reduced in spatial dimension, followed by an expansive path, in which the feature maps were expanded back to their original size. The contractive path consisted of convolutional blocks alternating with maxpool (downsampling) operations with 2 × 2 kernel and stride 2. Each convolutional block consisted of two 3 × 3 convolutions, each followed by a rectified linear unit activation. The number of output channels of the convolutions started at 32 and doubled after each downsampling step. There were 5 total convolutional blocks in the contractive path, alternated with 4 downsampling layers. The expansive path consisted of 4 steps, each consisting of 2× upsampling by bilinear interpolation, concatenation with the downsampling remainder of the corresponding level of the contractive path, and a convolutional block as above. In the concatenation step, we concatenated the remainder, which can be written as

**Figure 1:**
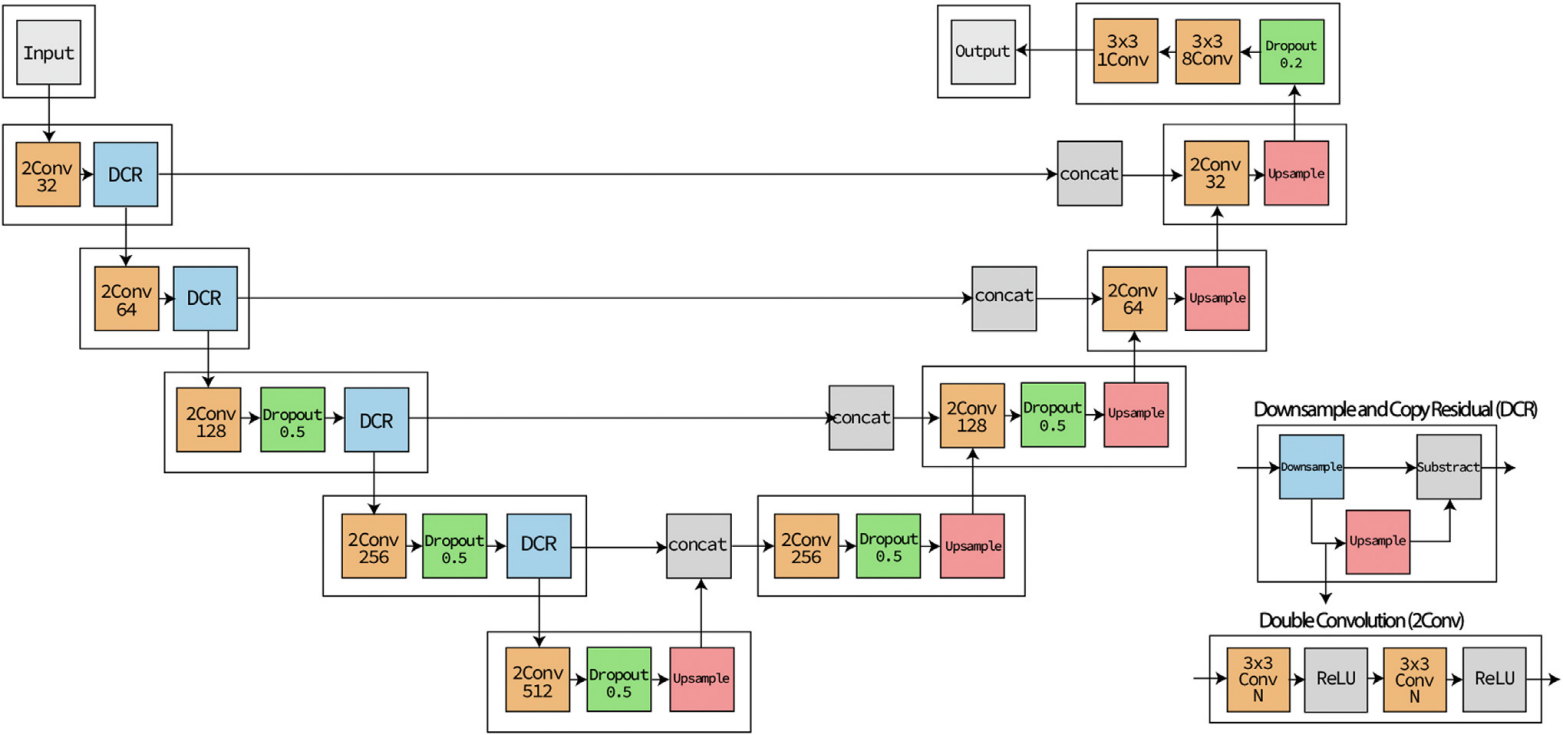
Diagram of the segmentation network. Green dropout operations are only present in UNet-Drop. Downsample and copy residual operation is expanded in the inset. ReLU = rectified linear unit.

(1) rem(x)=x−upsample(downsample(x))

where “upsample” is the 2× bilinear upsampling and “downsample” is the 2 × 2 maxpool operation. After the expansive path, there was a dropout layer with drop probability 0.2 and 2 final convolution layers. The penultimate convolution used a 3 × 3 kernel with 8 output channels and was followed by a rectified linear unit activation; the final convolution used a 3 × 3 kernel with 1 output channel and had a final activation function, which can be written as

(2) $$final\left(x\right)=\frac{1}{2}\left(\frac{x}{\sqrt{x^{2}+1}}+1\right)$$

and which ensured the output was between 0 and 1. For the UNet-Drop models, an additional dropout layer with drop probability 0.5 was added after the last 3 convolutional blocks of the contractive path and the first 2 convolutional blocks of the expansive path, as shown in Figure 1. Dropout was also left on during inference.

Models were trained with the Adam optimizer with an initial learning rate of 0.0001. This rate was multiplied by 0.999 each epoch. Each model was trained for 5000 epochs. Dice loss (1 minus the Dice coefficient) was used as the loss function. Hoyer-Square regularization^14^ was added to the loss with a coefficient of 0.001. Data augmentation was applied as in Pramil et al.^11^

### Model Specifications

A total of 6 models were trained. For the first group of models, we initially trained 5 UNet models with the architecture as described above with fivefold cross validation. The dataset was split into 5 equal partitions and each of the models was validated on 1 of those partitions while training on the other 4 partitions. The 5 separate models trained this way were labeled UNet-1, UNet-2, UNet-3, UNet-4, and UNet-5. The output probabilities from these 5 models were averaged to create UNet-Avg (ensemble method). The UNet-1 model was used as a baseline comparison as well as used for computing the UNet-Avg results.

The final model trained was the dropout model which used the same UNet architecture except with the additional dropout layers as specified previously (dropout method). The dropout model outputs were named after the number of times they were run for inference on the same image. UNet-Drop4 was the average of 4 runs of inference from the dropout model, UNet-Drop8 was the average of 8 runs and so on. UNet-Drop64, which was the average of 64 runs of inference on the same image, is referred to as UNet-Drop for the remainder of the manuscript.

### Uncertainty Measurement

Uncertainty was calculated using the formula for Shannon Entropy, which is a method for quantifying the degree of uncertainty in a prediction.^15^ We calculated entropy using the final outputs after passing through the activation function, Eq. (2), for the GA class. The relationship between the final outputs, which is interpreted as a probability, and entropy is described in Figure S2 (available at www.ophthalmologyscience.org). Entropy is greatest when the probability of GA for a given pixel is 0.5. Entropy was calculated with the following formula:

(3) $$Entropy=-\sum_{i=1}^{N}p\left(x_{i}\right)\log_{2}\left(p\left(x_{i}\right)\right)$$

where N is the number of classes (2) and p(x_i_) is the probability that the pixel belongs to the class i.

The UNet-1 through UNet-5 models and various iterations of the dropout model were evaluated by generating outputs on images from the held-out test set. All the subsequent model results including UNet-Avg and UNet-Drop were calculated by averaging over different model outputs. We used a sliding threshold of entropy to calculate Dice scores with entropy thresholds starting at <1% and increasing by 10% all the way to 100%. We identified all the pixels from a model's prediction that were at or below the set entropy threshold, then calculated 2 Dice scores: one comparing the model's predictions and grader 1 and the second between the model's predictions and grader 2. The final reported Dice for a given model at a given entropy was the average between those 2 scores. The percentage of the image removed was calculated by identifying all the pixels in an image above a given entropy threshold, and dividing that number of pixels by the total number of pixels in the image.

### Statistical Tests

Significance between the Dice scores was assessed using Wilcoxon signed rank test. Significance levels were adjusted with the Bonferroni correction for multiple comparisons. Pearson correlations were run to find the correlation between Dice scores and percentage of the image removed.

### Software and Hardware

The software packages and versions used for this manuscript were as follows: python 3.10; pytorch 1.11; and cuda 10.2. We used 8 Nvidia P-100 graphics processing units for training and inference.

## Results

**Figure 3:**
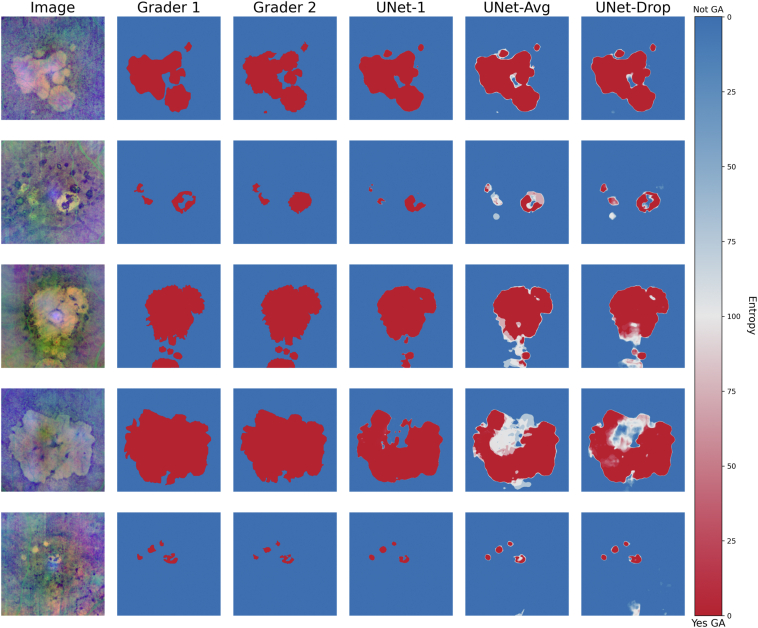
Representative images from the dataset with corresponding grader segmentations and outputs from UNet-1, UNet-Avg, and UNet-Drop. The entropy is displayed with a sliding color scale. Increasingly darker red color represents where the models are certain there is GA and increasingly darker blue color represents where the models are certain there is no GA. GA = geographic atrophy.

Example segmentations from the test set are shown in Figure 3. Qualitatively, both UNet-Avg (ensemble method) and UNet-Drop (dropout method) output a greater number of pixels with high entropy when compared with UNet-1. When compared with each other, UNet-Avg and UNet-Drop have different levels of entropy at any given pixel from an input image. In many cases, such as the fourth row, UNet-1 makes segmentation mistakes when compared with the ground truth of grader 1 and grader 2 but is highly confident about its segmentation; UNet-Avg and UNet-Drop are also incorrect but are less confident and have greater entropy in the areas of incorrect pixels. Pixels at the border of the GA lesions seem to have greater entropy in both UNet-Avg and UNet-Drop. An alternate plot of Figure 3 as posterior probabilities or model certainties of GA is given in Figure S4 (available at www.ophthalmologyscience.org).

**Figure 5:**
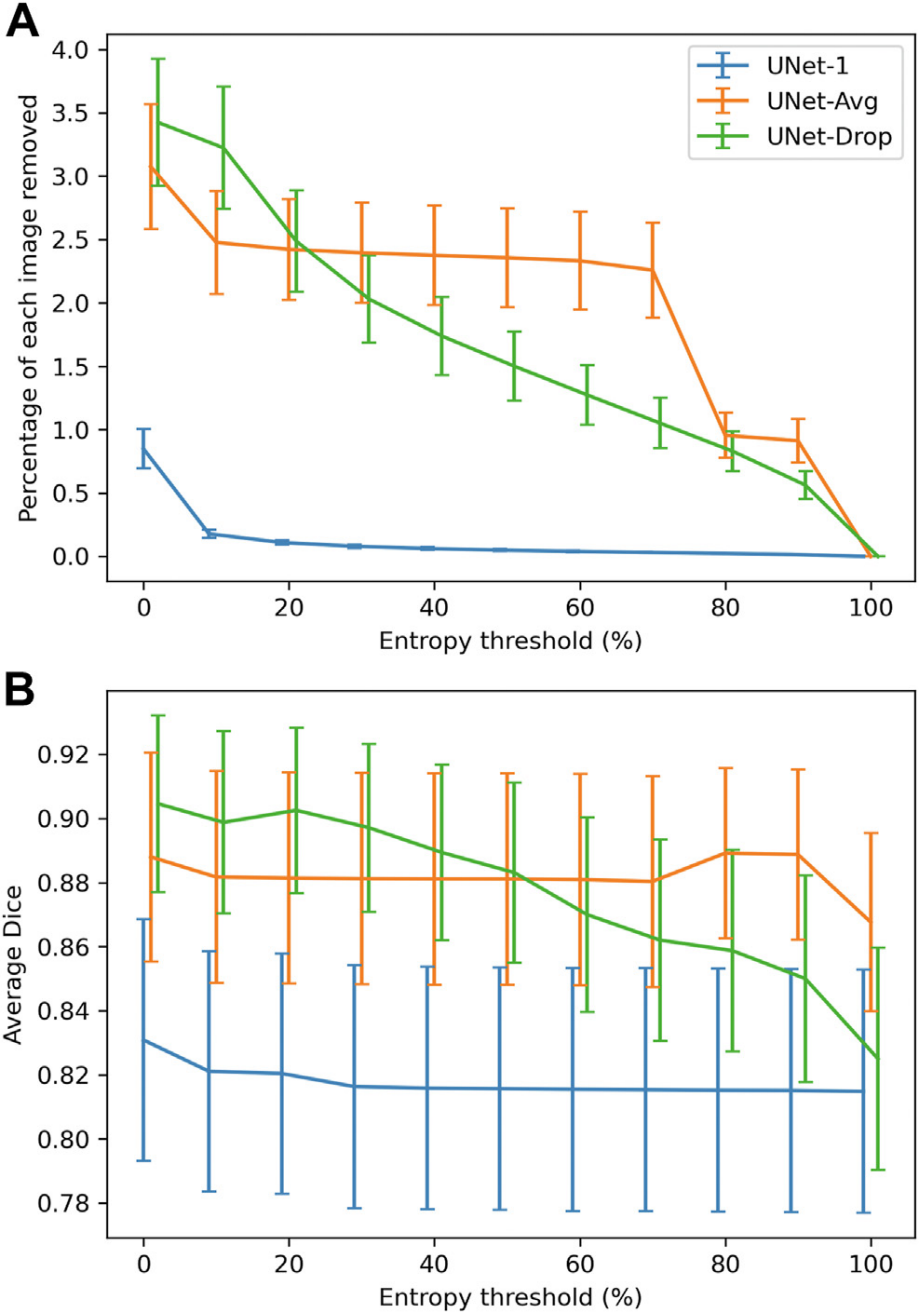
**A,** The percentage of each image removed when using a given entropy threshold for models UNet-1, UNet-Avg, and UNet-Drop. **B,** Dice scores achieved when removing pixels in the image above a certain entropy threshold for the models.

The average percentage of each image removed for being over the uncertainty threshold is shown in Figure 5A. At an entropy threshold of 10%, the percentage of each image removed for UNet-1, UNet-Avg, and UNet-Drop were 0.2 (0.1–0.2) percent, 2.5 (2.1–2.9) percent, and 3.2 (2.7–3.7) percent respectively, indicating that UNet-Drop and UNet-Avg had a greater percentage of uncertain pixels in their prediction than UNet-1. As the entropy threshold increases and more uncertain pixels are included in the segmentation, a smaller percentage of the image is removed.

Quantitatively, the average Dice scores on the test set for each network and each entropy threshold are shown in Figure 5B. UNet-Avg and UNet-Drop both produce higher Dice scores than UNet-1, especially with lower entropy thresholds. Using an entropy threshold of 10%, the Dice scores of UNet-1, UNet-Avg, and UNet-Drop were 0.82 (95% confidence interval 0.78–0.86), 0.88 (0.85–0.91), and 0.90 (0.87–0.93), respectively. The Dice scores for UNet-Avg and UNet-Drop were significantly higher than the scores for UNet-1 according to the Wilcoxon signed rank test (*P* < 0.001).

**Figure 6:**
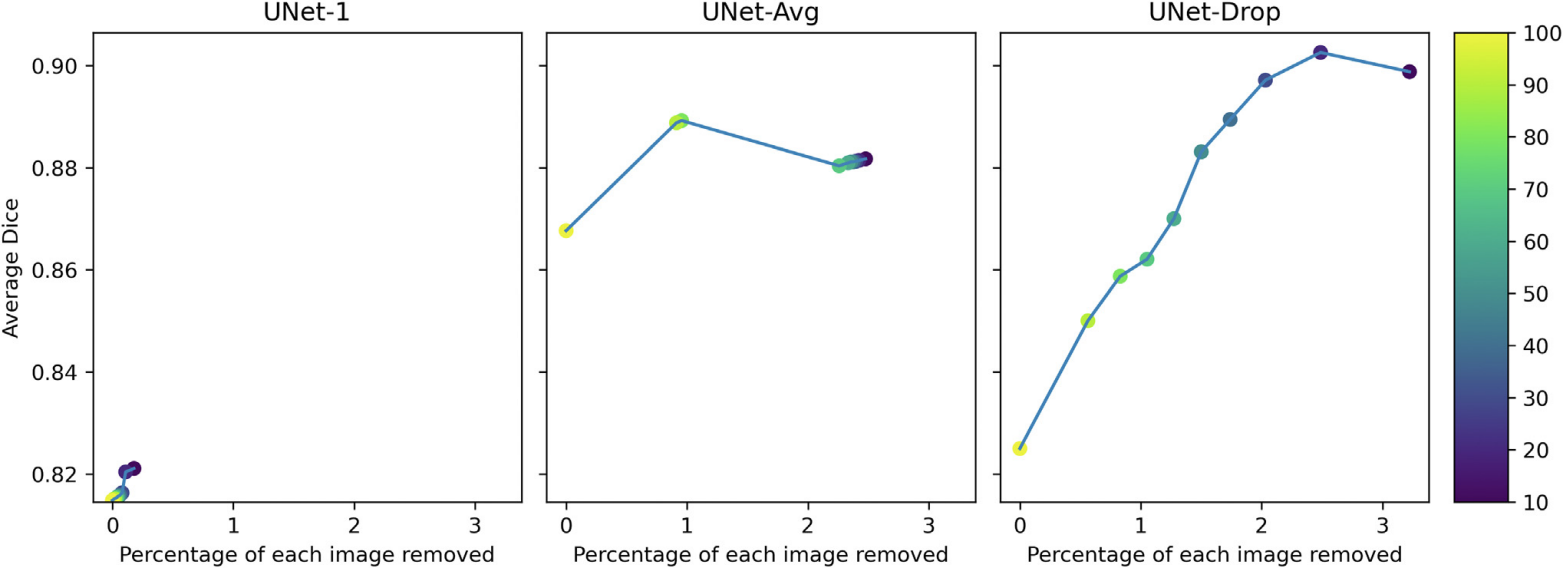
Tradeoff between percentage of each image removed and average Dice for UNet-1, UNet-Avg, and UNet-Drop. Color denotes entropy threshold.

A direct comparison of Dice score versus the percentage of each image removed is shown in Figure 6. As the percentage of the image removed increased, the Dice score also increased. There was a strong correlation between Dice score and percentage of each image removed for UNet-1 and UNet-Drop: Pearson rho 0.931 (*P* < 0.05) for UNet-1, 0.931 (*P* < 0.05) for UNet-Drop, and 0.232 (*P* = 0.519) for UNet-Avg.

Average Dice scores and percentage of each image removed are shown for UNet-Drop4 through UNet-Drop64 in Figure S7 (available at www.ophthalmologyscience.org). UNet-Avg is also shown for comparison. Running the dropout network more times per image generally improved performance. There was a statistically significant difference between the average Dice scores of UNet-Drop4 and UNet-Drop64 at an entropy threshold of 10% (*P* < 0.05/15). Time taken to run inference on the entire test set is shown in Table S1 (available at www.ophthalmologyscience.org).

## Discussion

We implemented and compared 2 Bayesian methods for estimating the uncertainty associated with GA lesions segmentation in pseudo-color SS-OCT en face images. Both UNet-Drop, a Monte Carlo dropout method, and UNet-Avg, an ensemble method, gave broader measures of epistemic uncertainty than the naive method of using a single model’s (UNet-1) final output.

The uncertainty from these Bayesian methods yields valuable insights into the models' thought process. This is illustrated in Figure 3, where the Bayesian methods show high entropy where there is corresponding uncertainty among the graders. For example, in the first row, grader 1 and grader 2 disagree on the small nodule in the top middle, and UNet-Avg and UNet-Drop have high entropy. Similarly, in the second row, the graders disagree on the GA area to the right middle, and the Bayesian models affirm this uncertainty through their high entropy in that region. Moreover, when UNet-Avg and UNet-Drop inaccurately segment GA, as in the central area of the fourth row, their entropy for these misclassified pixels is close to 100% indicating that both models are highly uncertain. In contrast, UNet-1 is overconfident in its predictions, even when incorrect. For example, in the image displayed on the second row, UNet-1 incorrectly labels the area on the top right of the largest lesion as GA absent with 0% entropy, even though the human graders labeled that area as GA present. This phenomenon has been well described previously, and it is hypothesized to be due to overfitting.^16^^,^^17^

In addition, the Bayesian models of UNet-Avg and UNet-Drop, achieved higher Dice scores than the traditional deep learning model UNet-1 with the same amount of training data. Furthermore, we demonstrated that as pixels with high entropy are removed, the Dice score of the Bayesian models increases. In particular, UNet-Drop has both the strongest correlation between the percentage of each image removed and Dice score, and the highest Dice score. Therefore, entropy thresholds could be used as a threshold to improve the performance of a deep learning model.

Not only did the Bayesian methods achieve higher Dice scores than the standard UNet-1, but they also provided transparency into their decisions through the easily interpretable uncertainty measures for segmented areas. Transparency is one of the key principles of “trustworthy” AI, i.e., AI systems that are safe, fair, and reliable.^18^ For clinicians to trust AI devices, they should be able to understand how sure the model is about its “diagnosis.” Ideally, a GA segmentation model would accurately identify areas of GA on SS-OCT images with high certainty, but it can be impractical to train such a model. First, it is challenging to accurately segment the presence or absence of disease features with traditional supervised segmentation models such as UNet-1, especially if the training dataset is small and not representative of disease complexity. Augmenting the training data can improve model performance but would require labor-intensive manual segmentations that may not be feasible. Furthermore, given disagreements among clinical experts, it would often require manual labeling by multiple experts to provide diversity in labels. Second, the traditional deep learning models are overconfident about their predictions for both the presence and absence of disease features.^17^ This overconfidence is intrinsic to their architecture, and these models will still exhibit overconfidence even with more training data.^19^ For example, in Figure 3, UNet-1 was so overconfident in its predictions that the only visible area of uncertainty among all the examples was a small central region within the image on the fourth row. In contrast, the Bayesian models achieved higher Dice with the same amount of training data and provided uncertainty measures of its predictions. These uncertainty measures can be overlaid on the predictions and be presented to clinicians for review, thereby increasing transparency into these models. Therefore, the Bayesian deep learning models are practical tools to incorporate deep learning models with uncertainty in both research and clinical practice.

The uncertainty estimates described in this manuscript have many clinical implications. First, the Bayesian methods allow one to calculate the expected area of GA. In addition, clinicians could use a model's uncertainty to aid their own clinical judgment. They could look at the prediction output and the entropy to use the AI model as a probabilistic measure, and trust outputs where the model has low entropy and is confident but place less trust on predictions with high entropy values. In clinically deployed algorithms, uncertainty could be used as a safety measure to understand how confident a model is in its predictions when applied to a new dataset.^20^ Next, entropy can be used as a threshold to improve segmentation performance as demonstrated in Figures 5 and 6. An entropy threshold could be used, and the model only shows segmentations that meet the threshold. In a future application, a clinician could also adjust the entropy threshold while examining a model's output to further guide their clinical decision-making.

These methods provide a rigorous system for estimating uncertainty of automated GA segmentation. There are limitations to this approach, including that it is computationally expensive, and our test set was relatively small. In addition, these results are only applicable to this unique task, such as identifying GA on images obtained from a particular OCT device. However, these methods for estimating uncertainty should generalize to similar problems as the dropout and ensemble training paradigms are agnostic of the input data. We did not try any additional model architectures other than UNet in our study. Another limitation is that uncertainty estimation is a broad field and there are many different models and training paradigms that have been previously described. We were limited by computational cost and training time, and future work is required to evaluate additional methods to calculate uncertainty. Lastly, we did not see if these methods improved clinical performance or decision-making.

This paper serves as an introduction of the concept of uncertainty. Estimating uncertainty from the predictions of deep learning models has many practical applications around understanding a model's confidence, and clinicians may use uncertainty to better understand which predictions from a model are more or less likely to be accurate. Future work is required to determine if these methods improve clinicians' trust in model predictions.
