# Supplementary material for: Estimating Uncertainty of Geographic Atrophy Segmentations with Bayesian Deep Learning
Source: Ophthalmol Sci. 2024 Jul 24;5(1):100587. doi: 10.1016/j.xops.2024.100587 (PMC11459066; doi:10.1016/j.xops.2024.100587)
Supplement: Supplemental Table 1 [file mmc1.pdf]

Supplemental Table 1.Time in seconds used to perform inference on the test set (containing 225 images) using a GeForce RTX 2080 Ti

Model	Time (s)
UNet-1	8
UNet-Avg	40
UNet-Drop4	15
UNet-Drop8	25
UNet-Drop16	44
UNet-Drop32	87
UNet-Drop64	172

UNet-Avg time is inferred by multiplying UNet-1 time by 5
