# Supplementary material for: Estimating Uncertainty of Geographic Atrophy Segmentations with Bayesian Deep Learning
Source: Ophthalmol Sci. 2024 Jul 24;5(1):100587. doi: 10.1016/j.xops.2024.100587 (PMC11459066; doi:10.1016/j.xops.2024.100587)
Supplement: Figure S2 [file mmc2.pdf]

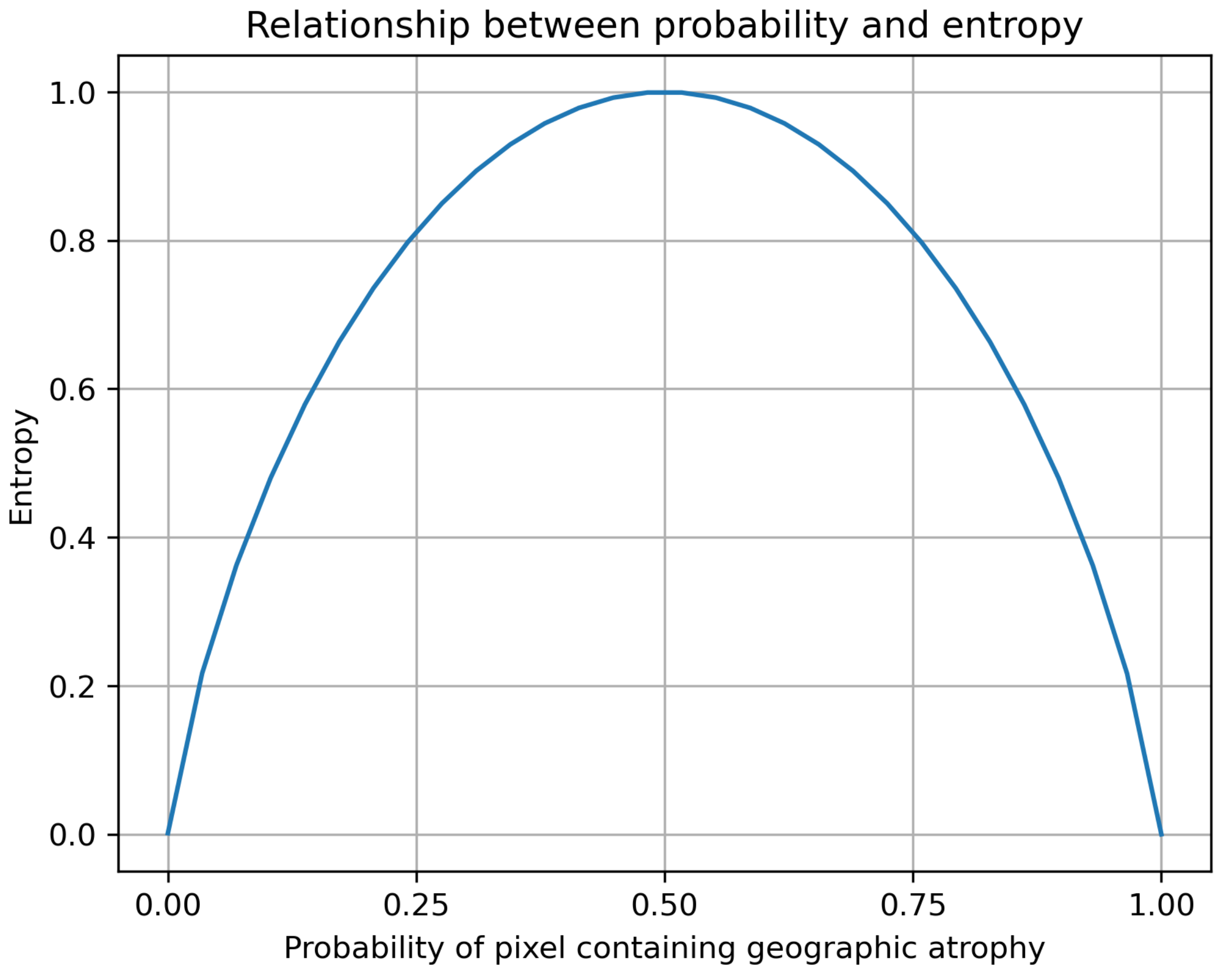

Supplemental Figure 1: Curve showing the relationship of the probability of a pixel containing geographic atrophy and the corresponding entropy value associated with it.
