# Supplementary figures and images for: Estimating Uncertainty of Geographic Atrophy Segmentations with Bayesian Deep Learning
Source: Ophthalmol Sci. 2024 Jul 24;5(1):100587. doi: 10.1016/j.xops.2024.100587 (PMC11459066; doi:10.1016/j.xops.2024.100587)

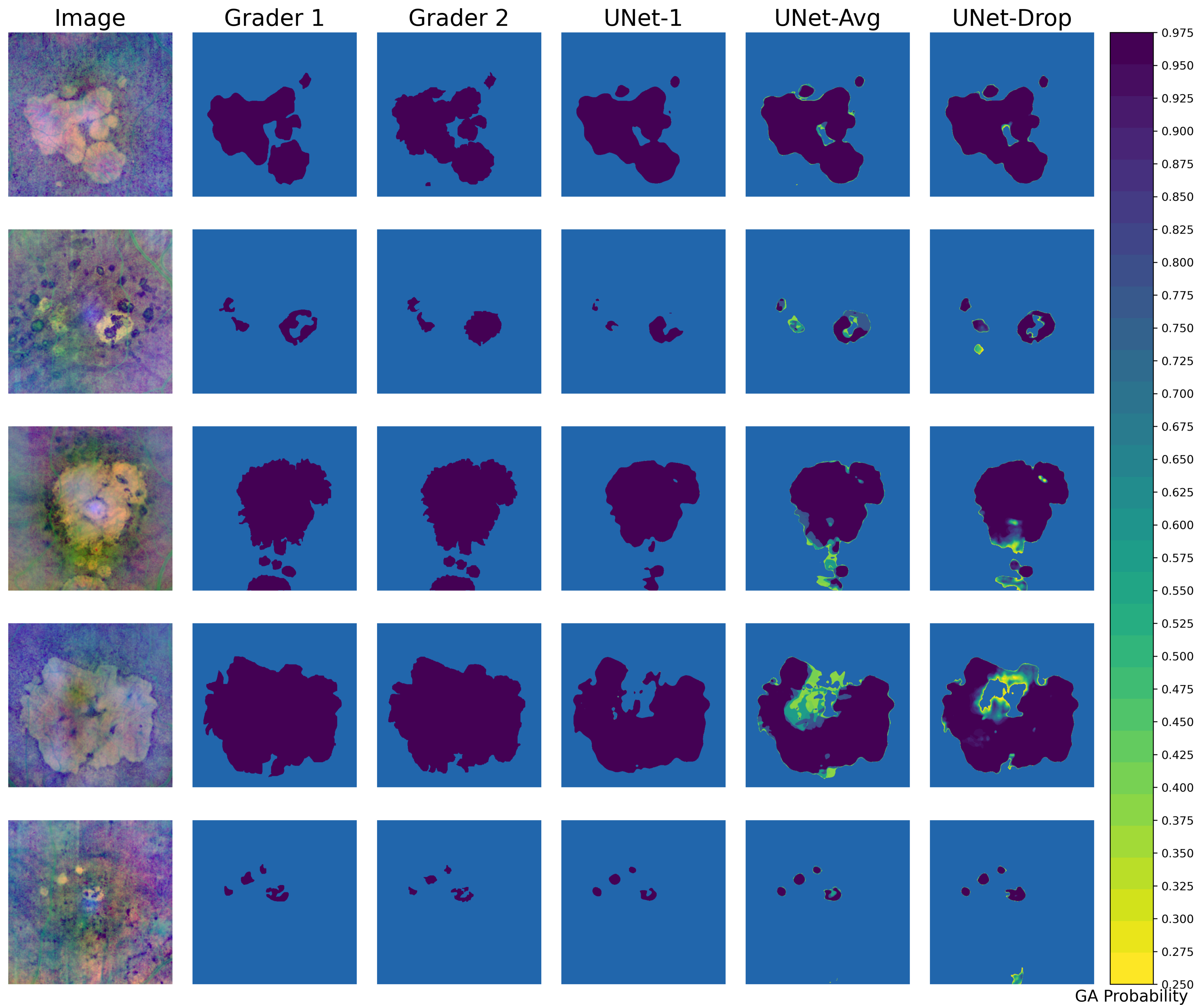

Supplement: Figure S4 [file mmc3.pdf]
