# Supplementary material for: Estimating Uncertainty of Geographic Atrophy Segmentations with Bayesian Deep Learning
Source: Ophthalmol Sci. 2024 Jul 24;5(1):100587. doi: 10.1016/j.xops.2024.100587 (PMC11459066; doi:10.1016/j.xops.2024.100587)
Supplement: Figure S7 [file mmc4.pdf]

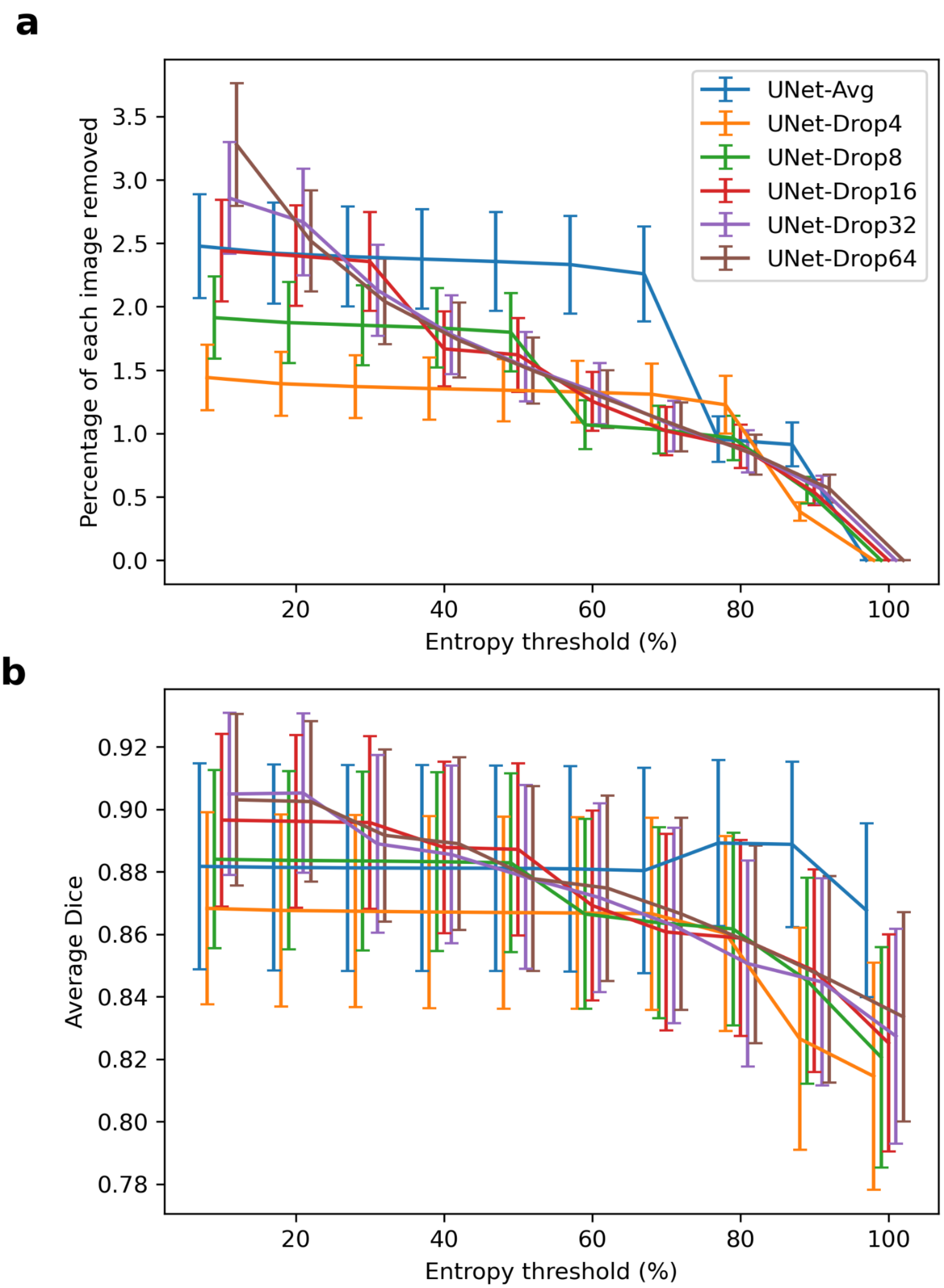

Supplemental Figure 2: Dice scores achieved when ignoring areas above given uncertainty thresholds, and percentage of each image ignored for UNet-Avg, and UNet-Drop averaging different numbers of outputs.
